# Detection of Sleep-Disordered Breathing Using Carbon Nanotube Sensors Predicts Complications After Lung Surgery

**DOI:** 10.1093/icvts/ivaf229

**Published:** 2025-09-25

**Authors:** Yasuhiro Nakashima, Masashi Kobayashi, Ayaka Asakawa, Katsutoshi Seto, Hironori Ishibashi, Shiro Sonoda, Tomoya Tateishi, Meiyo Tamaoka, Yasunari Miyazaki, Koshiro Okumoto, Haruka Horiuchi, Yoshikazu Nakajima, Kenichi Okubo

**Affiliations:** Department of Thoracic Surgery, Institute of Science Tokyo, Tokyo, 113-8519, Japan; Department of Thoracic Surgery, Tokyo Kyosai Hospital, Tokyo, 153-8934, Japan; Department of Thoracic Surgery, Institute of Science Tokyo, Tokyo, 113-8519, Japan; Department of Thoracic Surgery, Kurashiki Central Hospital, Okayama, 710-0052, Japan; Department of Thoracic Surgery, Institute of Science Tokyo, Tokyo, 113-8519, Japan; Department of Thoracic Surgery, Aichi Cancer Center, Nagoya, 464-8681, Japan; Department of Thoracic Surgery, Institute of Science Tokyo, Tokyo, 113-8519, Japan; Department of Respiratory Medicine, Institute of Science Tokyo, Tokyo, 113-8519, Japan; Department of Respiratory Medicine, Institute of Science Tokyo, Tokyo, 113-8519, Japan; Department of Respiratory Medicine, Institute of Science Tokyo, Tokyo, 113-8519, Japan; Department of Respiratory Medicine, Institute of Science Tokyo, Tokyo, 113-8519, Japan; Institute of Biomaterials and Bioengineering, Institute of Science Tokyo, Tokyo 113-8519, Japan; Institute of Biomaterials and Bioengineering, Institute of Science Tokyo, Tokyo 113-8519, Japan; Institute of Biomaterials and Bioengineering, Institute of Science Tokyo, Tokyo 113-8519, Japan; Department of Thoracic Surgery, Institute of Science Tokyo, Tokyo, 113-8519, Japan

**Keywords:** sleep-disordered breathing, carbon nanotube sensor, thoracic surgery, postoperative complications, air leak, respiratory monitoring

## Abstract

**Objectives:**

Sleep-disordered breathing significantly affects perioperative outcomes; however, it remains frequently undiagnosed. We aimed to evaluate the utility of a novel carbon nanotube sensor system for detecting postoperative breathing abnormalities and investigated its association with postoperative complications following thoracic surgery.

**Methods:**

In this prospective study, 86 patients who underwent anatomical lung resection without previously diagnosed obstructive sleep apnoea were monitored using carbon nanotube sensors from the immediate postoperative period through the first postoperative day. Abnormal breathing was defined as an ≥30% reduction in the peak sensor signal from baseline lasting more than 10 s, in accordance with standard hypopnea criteria used in polysomnography. Patient characteristics and complications were compared using Fisher’s exact and Mann-Whitney *U* test. Multivariate logistic regression identified predictors of major complications

**Results:**

Twenty-three patients (26.7%) exhibited abnormal breathing events (sleep-disordered breathing). This group had a higher proportion of males (87% vs 61.9%, *P* = .035), had more difficult intubation (42.1% vs 13.5%, *P* = .018), and more frequently received epidural anaesthesia in addition to general anaesthesia (65.2% vs 36.5%, *P* = .027). Multivariate analysis identified sleep-disordered breathing as an independent predictor of major complications (Clavien-Dindo grade ≥3; odds ratio 4.41, 95% CI 1.14-13.8, *P* = .011) and prolonged air leakage (odds ratio 15.6, 95% CI 2.39-102, *P* = .004).

**Conclusions:**

The carbon nanotube sensor showed potential for detecting undiagnosed sleep-disordered breathing after thoracic surgery, independently associated with increased risk of major complications, particularly prolonged air leakage.

**Clinical trial registration:**

UMIN-CTR (https://center6.umin.ac.jp/cgi-open-bin/ctr_e/ctr_view.cgi?recptno=R000035066). Trial number: UMIN000031533.

## Graphical abstract

**Figure:**
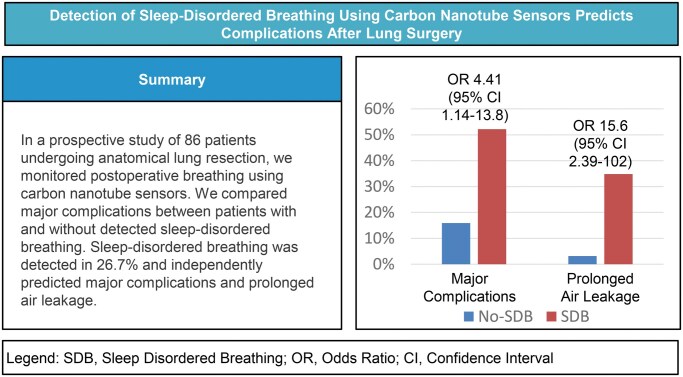

## INTRODUCTION

Traditional postoperative respiratory monitoring in thoracic surgery focuses primarily on oxygenation parameters, often overlooking ventilation pattern abnormalities such as sleep-disordered breathing (SDB). Obstructive sleep apnoea (OSA), the most common form of SDB, affects 14%-49% of middle-aged men and up to 50% of women.^1^ Given the high prevalence of OSA in patients with lung cancer,^2^ and the fact that more than 75% of patients with OSA remain undiagnosed or untreated,^1^ there is a clear need for screening and postoperative monitoring of SDB in thoracic surgery patients. Additionally, the limited availability of suitable monitoring devices has hindered research in this field, which suggests potential opportunities for improving patient care through enhanced detection methods.

Notably, more than 75% of patients with OSA remain undiagnosed or untreated.^1^ Surgical stress and anaesthesia can exacerbate existing OSA or trigger its onset in previously unaffected patients, with studies documenting new-onset postoperative OSA even in patients with normal preoperative overnight polysomnography.^3^ The American Society of Anesthesiologists guidelines emphasize that patients with confirmed or suspected OSA require enhanced perioperative care to minimize complications.^4^

However, monitoring of respiratory disorders after lung resection involves unique challenges. Standard polysomnographic monitoring tools, including airflow sensors and thoracic belts, are impractical owing to the need for supplemental oxygen administration and chest drain placement after surgery. Consequently, overnight polysomnography, the gold standard for OSA diagnosis, cannot be effectively utilized in the immediate postoperative period, limiting our understanding of the impact of SDB after lung cancer surgery.

In collaboration with the Yamaha Corporation, we developed a carbon nanotube (CNT) sensor system capable of monitoring chest wall movement during the perioperative period after lung resection. In this study, we aimed to evaluate the clinical utility of CNT sensors in detecting postoperative breathing abnormalities and investigate their association with postoperative complications in patients undergoing thoracic surgery.

## MATERIALS AND METHODS

### Patient selection

This prospective observational study enrolled patients who underwent anatomical lung resection (segmentectomy or lobectomy) for resectable lung tumours at Tokyo Medical and Dental University and Tokyo Kyosai Hospital between September 2018 and December 2021, with written informed consent obtained from all participants.

Patients with preoperatively diagnosed OSA and those with insufficient measurement duration (<6 h of valid data) were excluded from the analysis.

The study was approved by the Ethics Committees of Tokyo Medical and Dental University (M2017-255) in March 2018 and Tokyo Kyosai Hospital (2105001) in June 2021. This study was conducted in accordance with the STROBE (Strengthening the Reporting of Observational Studies in Epidemiology) guidelines and the Declaration of Helsinki (2013 revision), and was registered in the UMIN Clinical Trials Registry (Trial Number: UMIN000031533) prior to patient enrollment.

### CNT sensor system

**Figure 1.**
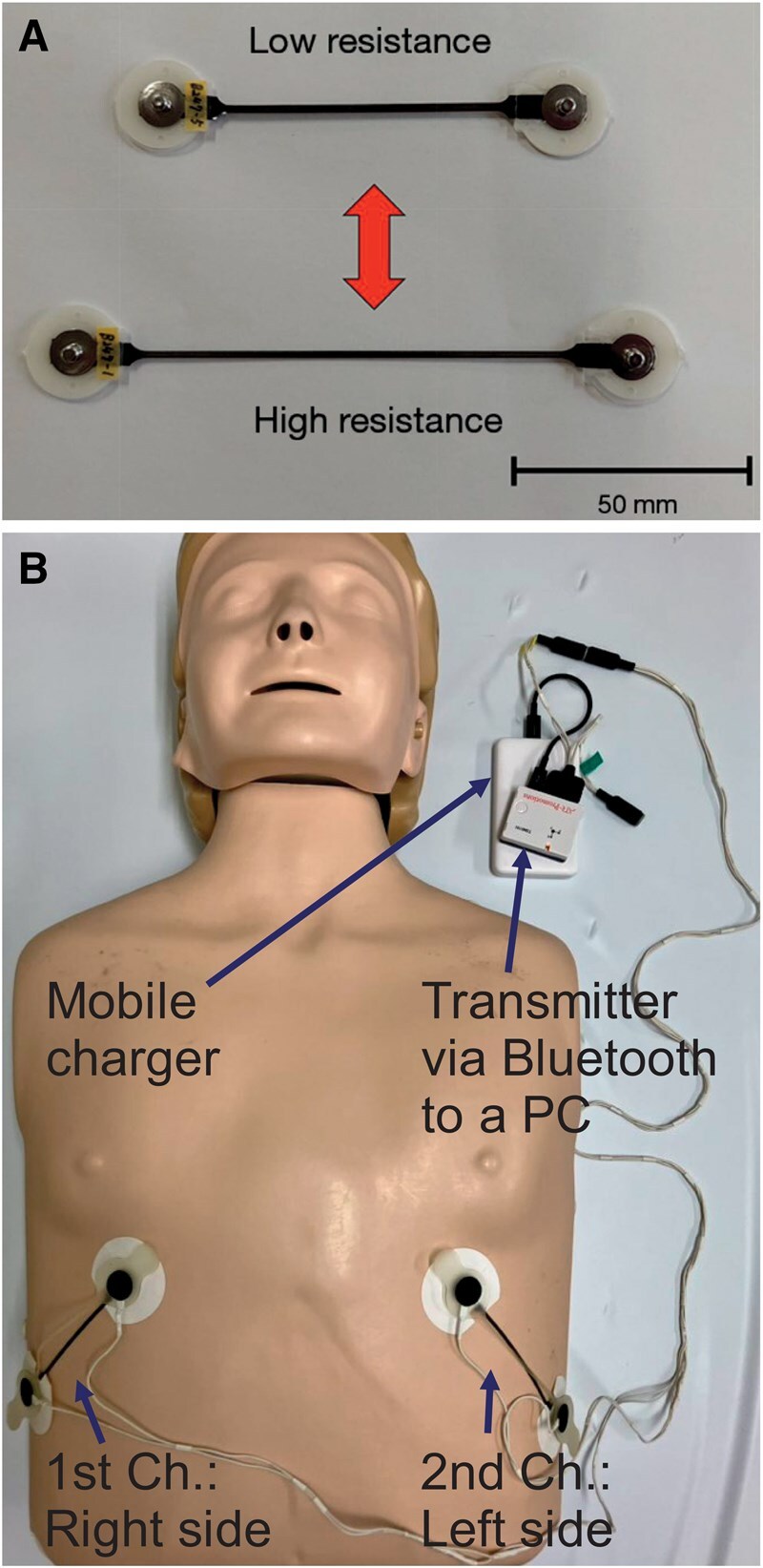
Carbon Nanotube (CNT) Sensor System. (A) The CNT strain sensor demonstrates different resistance levels based on elongation. The upper sensor shows a low resistance state, and the lower sensor shows a high resistance state. Scale bar: 50 mm. (B) Setup of the respiratory monitoring system. The CNT sensors are attached bilaterally to the chest wall, with the right side serving as the 1st channel and the left side as the second channel. The signal from each sensor is transmitted via Bluetooth to a personal computer through a mobile charger unit

The strain sensor system (Yamaha Corporation, Shizuoka, Japan) consisted of multi-wall CNTs, which are next-generation carbon materials composed of multiple layers of coaxial tubes. The sensor works by utilizing the semiconductor properties in the sheet section that enable the detection and recording of mechanical deformation through changes in electrical resistance. When the sheet is stretched or contracted, the nanoscale structure of the CNT changes, resulting in measurable changes in electrical resistance that correspond to the degree of deformation. This 6-cm-long, thin, flexible sheet demonstrated excellent response characteristics with up to 200% elongation capability and maintained linear changes in the elongation-resistance relationship (**Figure 1A**).

The CNT sensor sheets were attached bilaterally to the chest wall across the sixth-ninth ribs perpendicular to the rib direction using biocompatible adhesive sheets, creating a dual-channel configuration with sensors on the right (first channel) and left (second channel) sides of the chest (**Figure 1B**). This placement allowed the sensor’s elongation to synchronize directly with respiratory chest wall movements. The strain sensor sheets were connected via wire to a transmitter and a mobile charger unit. The collected sensor data were transmitted via Bluetooth to a personal computer equipped with analysis software for real-time analysis and recording. Raw sensor data were processed using a finite impulse response filter to remove non-respiratory artefacts, including positional changes and transient muscle contractions.^5^

### Postoperative monitoring protocol

The CNT sensor system was attached to bilateral chest walls within 1 h after surgery completion upon admission to the surgical intensive care unit, and monitoring was initiated. On the operative side, chest tubes are typically inserted along the anterior axillary line; therefore, the CNT sensor was positioned slightly posterior to the chest tube. CNT sensor signals were continuously recorded from immediately after surgery until 8:00 AM the following morning. Patients followed standard postoperative care protocols, remaining on bed rest with head elevation permitted until the following morning, and routine position changes were allowed as per standard practice.

### Respiratory waveform analysis

**Figure 2.**
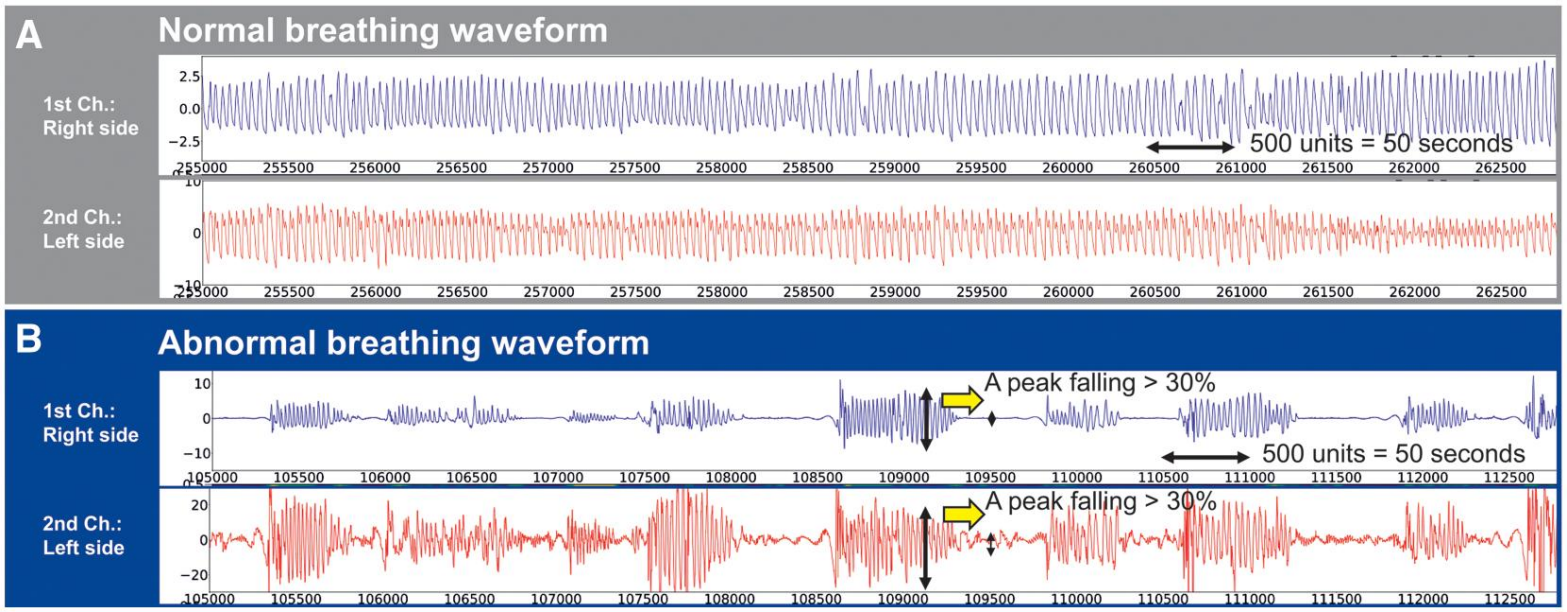
Respiratory Waveforms Recorded by Carbon Nanotube (CNT) Sensors. The upper blue line shows the right side (first channel), and the lower red line shows the left side (second channel). One unit on the graph corresponds to 0.1 s, Resulting in a total duration of 50 s for every 500 units displayed. (A) Normal breathing pattern showing regular, periodic waveforms observed on both sides. (B) Abnormal breathing pattern with yellow arrows indicating peak signal drops of more than 30% from baseline, representing abnormal breathing events observed simultaneously on both sides

Normal breathing patterns were characterized by regular periodic oscillations with consistent amplitude and frequency (**Figure 2A**), while abnormal breathing was characterized as fluctuating respiratory waveforms showing alternating patterns between normal breathing and abnormal events (**Figure 2B**). An abnormal breathing event was defined as a peak signal waveform dropping by ≥30% from the baseline and lasting for ≥10 s (**Figure 2B**). This definition reflected the American Academy of Sleep Medicine (AASM) Scoring Manual criteria for hypopnea using nasal pressure in polysomnography measurements.^6^ According to the AASM criteria, hypopnea is scored when all of the following criteria are met: (1) peak signal excursions drop by ≥30% from the pre-event baseline using nasal pressure, (2) the duration of the ≥30% drop in signal excursion is ≥10 s, and (3) there is a ≥3% oxygen desaturation from the pre-event baseline and/or the event is associated with an arousal. Due to routine postoperative oxygen supplementation, oxygen desaturation changes were excluded from the definition of abnormal breathing events. Patients were classified into the SDB group when they exhibited sustained patterns of alternating abnormal breathing events and normal breathing cycles, with a minimum threshold of 15 or more alternating cycles during the monitoring period. To ensure reproducibility, brief non-periodic abnormal breathing patterns containing sudden high-amplitude spike waveforms indicative of movement artefacts were excluded. All respiratory waveforms were independently evaluated by 2 board-certified thoracic surgeons (Y.N. and M.K.) who were blinded to patient outcomes. Disagreements between the 2 evaluators (occurring in <5% of cases) were resolved through team discussion to determine the final classification.

### Perioperative data analysis

#### Clinical data collection

Hospital records were reviewed to assess patient characteristics, surgical details, and postoperative outcomes. Complete clinical and outcome data were available for all 86 patients included in the analysis, with no missing variables.

#### Complication assessment

Postoperative complications within 30 days after surgery were classified according to the Clavien-Dindo classification.^7^ Respiratory complications were defined as atelectasis, mucus plugging, and postoperative pneumonia. Air leak complications were categorized under prolonged air leak and excluded from the respiratory complications category to avoid overlap.

#### Statistical analysis

Analyses were performed using EZR version 1.66 based on R version 3.4.1.^8^ For the comparison between the groups without SDB (no-SDB) and with SDB (SDB), univariate analysis of demographic and clinical variables was performed, including sex, age, body mass index, smoking history, presence of emphysema, or interstitial pneumonia (IP) on CT images, preoperative respiratory function, Cormack classification after laryngoscopy, use of combined epidural and general anaesthesia, surgical method, surgical approach, video-assisted thoracoscopic surgery, operative time, and postoperative complications. Fisher’s exact test was used for categorical variables, and the Mann-Whitney *U* test for continuous variables. For multivariate analysis, variables with *P* < .15 in univariate analysis were initially included, along with age and sex as clinically important adjusters regardless of univariate significance. Multivariate logistic regression was performed using the backward elimination method with removal criteria of *P* > .10. The backward elimination process identified CT findings of emphysema and SDB as statistically significant independent predictors. Age and sex were retained in the final model as standard demographic adjusters.

## RESULTS

### Patient characteristics

After excluding 2 patients with preoperatively diagnosed OSA, 96 patients underwent CNT sensor monitoring. Ten patients (10.4%) were subsequently excluded due to insufficient measurement duration (<6 h of valid data), primarily due to inadequate sensor attachment (*n* = 8) and sensor detachment from perspiration (*n* = 2), leaving 86 patients for final analysis.

All procedures were performed under general anaesthesia with endotracheal intubation. Surgical approaches included video-assisted thoracoscopic surgery in 58 patients (67.4%) and open thoracotomy in 28 patients (32.6%). Anatomical resections comprised segmentectomy in 19 patients (22.1%) and lobectomy or greater resection in 67 patients (77.9%). Postoperative pain management included epidural anaesthesia with ropivacaine in 38 patients (44.2%) and continuous subcutaneous fentanyl infusion in the remainder, with all patients receiving standardized multimodal analgesia protocols. Notably, the SDB group demonstrated significantly higher rates of difficult airways (Cormack score > 1) and more frequent use of epidural anaesthesia (both *P* < .05) (**Table 1B and C**).

**Table 1.** Clinical Characteristics

Characteristics	All patients (*n* = 86)	No-SDB (*n* = 63)	SDB (*n* = 23)	*P*-value^a^
**A. Patient characteristics**				
Age (years) median (range)	71 (38-88)	71 (38-88)	73 (49-82)	.820
Sex, *n* (%)	Male = 59 (68.6%)	Male = 39 (61.9%)	Male = 20 (87%)	.035
	Female = 27 (31.4%)	Female = 24 (38.1%)	Female = 3 (13%)	
BMI ≥ 25, *n* (%)	24 (27.9%)	17 (27%)	7 (30.4%)	.789
Smoking, *n* (%)	66 (76.7%)	45 (71.4%)	21 (91.3%)	.082
Heavy smoker	12 (14.0%)	9 (14.3)	3 (13%)	1.000
Moderate-heavy smoker	53 (61.6%)	36 (57.1%)	17 (73.9%)	.212
CT findings (emphysema, IP, or CPFE), *n* (%)	42 (48.8%)	17 (37.8%)	25 (61.0%)	.051
CT findings (emphysema), *n* (%)	29 (33.7%)	20 (31.7%)	9 (39.1%)	.609
%VC, median (range)	102.7 (42.6-137.0)	100.3 (53.2-129.7)	113.5 (42.6-137.0)	.036
FEV1%, median (range)	72.3 (34.8-96.19)	72.64 (34.8-96.19)	70.80 (45.6-94.20)	.707
%FEV1, median (range)	88.0 (42.8-119.5)	87.4 (42.8-117.5)	88.6 (46.4-119.5)	.911
%DLCO, median (range)	94.4 (40.8-167.2)	97.4 (44.8-167.2)	87.0 (40.8-128.3)	.084
Pulmonary dysfunction, *n* (%)	42 (48.8%)	31 (49.2%)	11 (47.8%)	1.000
**B. Perioperative characteristics**				
Cormack >1, *n* (%)	15 (21.1%)	7 (13.5%)	8 (42.1%)	.018
Epidural anaesthesia	38 (44.2%)	23 (36.5%)	15 (65.2%)	.027
Anaesthesia time, minutes, median (range)	233 (150-414)	233 (150-412)	258 (169-414)	.315
IntraOp-pCO_2_ > 45, *n* (%)	44 (57.1%)	27 (48.2%)	17 (81%)	.011
**C. Surgical procedure**				
Surgery time, minutes, median (range)	158 (69-344)	155 (69-320)	186 (109-344)	.283
Extent of resection: Lobectomy and more, *n* (%)	67 (77.9%)	48 (76.2%)	19 (82.6%)	.770
Surgical approach: VATS, *n* (%)	58 (67.4%)	46 (73%)	12 (52.2%)	.076

a The Mann-Whitney *U* test was used for continuous variables, and Fisher’s exact test was used for categorical variables.

Abbreviations: BMI: body mass index; CPFE: combined pulmonary fibrosis and emphysema; DLco: diffusing capacity of the lung for carbon monoxide; FEV1: forced expiratory volume in 1 second; IP: interstitial pneumonia; *n*: number of patients; SDB: sleep-disordered breathing; VATS: video-assisted thoracoscopic surgery; VC: vital capacity.

Of the 86 enrolled patients, 23 (26.7%) exhibited abnormal respiratory events on CNT sensors (SDB group). While we initially detected waveforms showing ≥30% amplitude reduction lasting more than 10 s, after excluding those with movement artefacts or irregular breathing patterns, all respiratory events classified as abnormal in the final analysis demonstrated amplitude reductions lasting more than 20 s. The median age was 71 years (range: 38-88), with a significant male predominance in the SDB group (**Table 1A**). While most baseline characteristics showed no significant differences between the groups, vital capacity (%) differed significantly.

The SDB group demonstrated significantly higher rates of difficult airways (Cormack score >1), epidural anaesthesia use, and elevated pre-extubation pCO_2_ (**Table 1B**). No significant differences were observed in any of the surgical parameters between the groups (**Table 1C**).

### Postoperative complications

Major complications (Clavien-Dindo grade ≥3) occurred in 22 patients (25.6%), with most respiratory complications developing within the first 48 h postoperatively. These included pleurodesis for prolonged air leak (*n* = 10, all cases with air leak onset by postoperative day 1), bronchoscopic intervention for atelectasis or mucus plugging (*n* = 10, diagnosed by postoperative day 2 with interventions on days 1-2), bronchoscopic intervention for haemorrhage-related atelectasis (*n* = 1), chest tube reinsertion for aspiration pneumonia (*n* = 1), and death due to acute exacerbation of IP on postoperative day 36 (*n* = 1). No major cardiac complications (Clavien-Dindo grade ≥3) were observed in either group.

The SDB group demonstrated significantly higher rates of major complications than the no-SDB group (**Table 2**). This difference was particularly evident in cases of prolonged air leak complications, all of which were managed with pleurodesis via the existing chest tubes. Notably, none of the patients required surgical intervention under general anaesthesia or developed complications requiring mechanical ventilation (grade ≥3b).

**Table 2.** Postoperative Complications

Characteristics	All patients (*n* = 86)	No-SDB (*n* = 63)	SDB (*n* = 23)	*P*-value^a^
Postoperative complication (grade^b^ ≥ 3), *n* (%)	22 (25.6%)	10 (15.9%)	12 (52.2%)	.002
Prolonged air leak (>5 days), *n* (%)	7 (8.1%)	1 (1.6%)	6 (26.1%)	.001
Prolonged air leak (grade^c^^b^ ≥3), *n* (%)	10 (11.6%)	2 (3.2%)	8 (34.8%)	<.001
Atelectasis or mucus plugging (grade^c^^b^ ≥3), *n* (%)	14 (16.3%)	8 (12.7%)	6 (26.1%)	.186
Atelectasis or mucus plugging (all grades^c^^b^), *n* (%)	20 (23.3%)	12 (19%)	8 (34.8%)	.153
Respiratory complication^d^^c^ (grade^c^^b^ ≥3), *n* (%)	15 (17.4%)	8 (12.7%)	7 (30.4%)	.104
Respiratory complication^d^^c^ (all grades^c^^b^), *n* (%)	27 (31.4%)	15 (23.8%)	12 (52.2%)	.018

a Fisher’s exact test or Mann-Whitney *U* test.

c Clavien-Dindo grade.

d Pneumonia, atelectasis, and mucus plugging.

Abbreviations: *n*: number of patients; SDB: sleep-disordered breathing.

Although atelectasis or mucus plugging alone showed no significant between-group differences, overall, respiratory complications (including pneumonia) were significantly more frequent in the SDB group.

### Risk factors for postoperative complications (Clavien-Dindo grade ≥ 3)

Univariate analysis identified 6 significant risk factors for postoperative complications (Clavien-Dindo grade ≥3): male sex, heavy smoking, moderate-heavy smoking, CT findings of emphysema/IP/combined pulmonary fibrosis and emphysema (CPFE), CT findings of emphysema, and SDB (**Table 3**). In multivariate analysis using backward elimination, CT findings of emphysema (OR 4.13, 95% CI 1.30-13.2, *P* = .017) and SDB (OR 5.94, 95% CI 1.80-19.6, *P* = .003) were identified as independent predictors of major complications (**Table 4**).

**Table 3.** Risk Factors of Postoperative Complications (Clavien-Dindo Grade ≥3)

Variable	Univariate		
	OR	95% CI	*P*-value^a^
Age ≥75 years	1.71	0.64-4.58	.289
Male	3.8	1.02-14.2	.047
Body mass index ≥25	0.958	0.32-2.84	.939
Heavy smoker	3.62	1.03-12.8	.045
Moderate-heavy smoker	3.73	1.13-12.3	.030
Smoker	3.91	0.83-18.5	.085
Pulmonary dysfunction (obstructive or restrictive)	2.25	0.83-6.11	.112
CT findings (emphysema, IP, or CPFE)	5.3	1.74-16.2	.003
CT findings (emphysema)	4.33	1.56-12.0	.005
VATS	1.05	0.37-2.95	.932
Epidural anaesthesia	1.75	0.66-4.66	.259
SDB	5.78	2.0-16.7	.001

a Logistic regression analysis.

Abbreviations: CPFE: combined pulmonary fibrosis and emphysema; IP: interstitial pneumonia; SDB: sleep-disordered breathing; VATS: video-assisted thoracic surgery.

**Table 4.** Risk Factors of Postoperative Complications (Clavien-Dindo Grade ≥3)

Variable	Multivariate		
	OR	95% CI	*P*-value^a^
Age ≥75 years	1.59	0.51-4.94	.426
Male	1.84	0.44-7.76	.408
CT findings (emphysema)	4.13	1.30-13.2	.0165
SDB	5.94	1.80-19.6	.0346

a Logistic regression analysis.

Abbreviation: SDB, sleep-disordered breathing.

### Risk factors for prolonged air leakage (Clavien-Dindo grade ≥ 3)

Univariate analysis revealed 3 significant risk factors for prolonged air leakage (Clavien-Dindo grade ≥3): heavy smoking, CT findings of emphysema, and SDB (**Table S1**). In multivariate analysis using backward elimination, CT findings of emphysema (OR 7.14, 95% CI 1.37-37.2, *P* = .020) and SDB (OR 19.1, 95% CI 3.28-111.0, *P* = .001) were identified as independent predictors of prolonged air leakage (**Table S2**).

### Risk factors for respiratory complications (all grades)

Univariate analysis identified significant predictors of respiratory complications: moderate-heavy smokers, current smokers, CT findings of emphysema/IP/CPFE, and SDB (**Table S3**). In multivariate analysis using backward elimination, moderate-heavy smoking (OR 5.68, 95% CI 1.44-22.5, *P* = .013) and SDB (OR 3.08, 95% CI 1.05-9.09, *P* = .041) were identified as independent predictors of respiratory complications (**Table S4**).

## DISCUSSION

The present study yielded 2 main findings. First, we discovered that CNT sensors could effectively detect latent abnormal breathing in 26.7% of patients without a prior diagnosis of OSA, suggesting the potential of CNT sensors for identifying postoperative abnormal breathing. Second, our results showed that SDB detected using CNT sensors was associated with a higher incidence of postoperative complications, particularly prolonged air leaks and respiratory complications, in patients undergoing lung resection surgery.

OSA, the most common type of SDB, affects 1%-9% of surgical patients, with rates rising to 24% when comprehensive screening questionnaires are used.^9^^,^^10^ Although the frequency of OSA in patients who have undergone lung resection remains unknown, its high prevalence of OSA among patients with lung cancer^2^ suggests that CNT sensors may be valuable for detecting postoperative breathing abnormalities. Postoperative sleep disturbance results from complex multifactorial interactions, including surgical stress, pain, opioid requirements, and environmental factors.^11^ Although anatomical factors contributing to upper airway obstruction and SDB were consistent with prior reports, our study revealed unanticipated findings regarding pain management strategies.^11^ Although the literature suggests epidural ropivacaine does not impair upper airway function,^12^ whereas systemic fentanyl may facilitate SDB, our observations contradicted these pharmacological expectations.^13^ This likely reflects institutional practice patterns favouring epidural anaesthesia for advanced malignancies requiring open thoracotomy, where disease-related factors may predominate over analgesic method.

Polysomnography remains the gold standard for diagnosing SDB; however, its complexity and resource requirements limit its routine perioperative use. Previous studies have investigated alternative measurement methods for breathing patterns, such as laser monitoring,^14^ real-time image sequences,^15^ and end-tidal CO_2_.^16^ CNT sensors offer several advantages particularly for perioperative measurements, including small size, lightweight design, ease of use even with chest drains in place on one side of the chest wall, and feasibility of evaluation despite oxygen administration. However, the pathophysiology of abnormal breathing detected by CNT sensors has not been established, making this elucidation a future challenge.

While no previous reports have focused on the perioperative outcomes of patients with coexisting OSA undergoing thoracic surgery, our findings align with those of studies of patients with OSA undergoing other surgical procedures. Previous studies on orthopaedic and general surgery have demonstrated increased perioperative adverse events in patients with OSA.^5^^,^^17^^,^^18^ OSA is associated with higher rates of postoperative hypoxemia, overall complications, and intensive care unit transfer.^9^^,^^17^ One proposed mechanism involves pharmacological and mechanical factors, such as neuromuscular blocking drugs and endotracheal tubes, which may disrupt the pharyngeal muscles and increase the aspiration risk.^18^ The American Society of Anesthesiologists guidelines emphasize the importance of preoperative screening and medical intervention for patients with OSA, recommending interventions such as adequate oxygen supplementation, non-supine positioning, minimizing the use of systemic opioids in favour of regional anaesthesia, and considering the postoperative continuous positive airway pressure.^4^ Our detection of abnormal breathing patterns in 26.7% of patients without prior OSA diagnosis, and their significant association with postoperative complications, suggests the clinical importance of undiagnosed SDB in thoracic surgical patients. However, whether these patterns represent pre-existing undiagnosed OSA, perioperative exacerbation of subclinical disorders, or transient stress-induced abnormalities remains unclear. If future studies confirm that abnormal breathing patterns detected by CNT sensors represent OSA-like pathophysiology, implementing guideline-recommended interventions in this previously unidentified patient population may improve perioperative outcomes.

Our study has several limitations that should be addressed in future research. First, this study was limited by the sample size and the restriction to 2 institutions within the same geographic area, which may have affected the generalizability of our findings. Second, the observation period was limited to the immediate postoperative period and continued until the following morning, which may not have captured all patients with abnormal sleep patterns. Future studies should implement multiple nights of monitoring spanning the preoperative and postoperative phases until discharge to provide a more comprehensive assessment of abnormal breathing patterns. Third, while CNT sensors demonstrate the potential for detecting abnormal breathing, validation against polysomnography has not been performed, and we cannot confirm the sensitivity and specificity of the detected abnormal breathing patterns in identifying true OSA versus other forms of SDB. Additionally, formal OSA evaluation and follow-up were not performed to determine whether patients with abnormal breathing were latent OSA patients. Finally, there may be potential confounding factors that were not considered in our study and could have influenced the results. These limitations highlight the need for larger and more comprehensive studies to further explore the utility of CNT sensors in perioperative care and interpret the clinical significance of our findings.

## CONCLUSION

The CNT sensor system shows potential for detecting abnormal breathing patterns in patients without prior OSA diagnosis, which may help identify high-risk patients who could benefit from SDB interventions. Further validation studies are needed to establish clinical utility and impact on patient outcomes.

## Supplementary Material

ivaf229_Supplementary_Data

## Data Availability

The data that support the findings of this study are available from the corresponding author upon reasonable request due to patient privacy considerations.
